# Recent development and future application of biodegradable ureteral stents

**DOI:** 10.3389/fbioe.2024.1373130

**Published:** 2024-03-20

**Authors:** Ke Hu, Zhipeng Hou, Yuanbin Huang, Xueying Li, Xiancheng Li, Liqun Yang

**Affiliations:** ^1^ Department of Urology, The Second Affiliated Hospital of Dalian Medical University, Dalian, China; ^2^ Research Center for Biomedical Materials, Engineering Research Center of Ministry of Education for Minimally Invasive Gastrointestinal Endoscopic Techniques, Shengjing Hospital of China Medical University, Shenyang, China; ^3^ College of Computer Science and Engineering, Dalian Minzu University, Dalian, China; ^4^ Liaoning Research Institute for Eugenic Birth and Fertility, China Medical University, Shenyang, China

**Keywords:** biodegradable ureteral stent, natural polymers, PLGA, PCL, PTMC, biodegradable metals

## Abstract

Ureteral stenting is a common clinical procedure for the treatment of upper urinary tract disorders, including conditions such as urinary tract infections, tumors, stones, and inflammation. Maintaining normal renal function by preventing and treating ureteral obstruction is the primary goal of this procedure. However, the use of ureteral stents is associated with adverse effects, including surface crusting, bacterial adhesion, and lower urinary tract symptoms (LUTS) after implantation. Recognizing the need to reduce the complications associated with permanent ureteral stent placement, there is a growing interest among both physicians and patients in the use of biodegradable ureteral stents (BUS). The evolution of stent materials and the exploration of different stent coatings have given these devices different roles tailored to different clinical needs, including anticolithic, antibacterial, antitumor, antinociceptive, and others. This review examines recent advances in BUS within the last 5 years, providing an in-depth analysis of their characteristics and performance. In addition, we present prospective insights into the future applications of BUS in clinical settings.

## 1 Introduction

Ureteral stents are widely used in the therapeutic management of urological diseases and are one of the most commonly used devices in clinical urology. Playing a crucial role in providing essential support and facilitating urine drainage, ureteral stents are routinely used in various clinical scenarios, including assisting in the treatment of urinary tract stones and alleviating and treating both benign and malignant ureteral obstructions. They are placed preoperatively to aid in intraoperative identification of the ureter, to facilitate expulsion of small residual stones in the upper urinary tract, and to prevent postoperative complications such as ureteral stricture (Lange et al., 2015; Wang et al., 2018).

In 1949, Herdman initially delineated ureteral stents constructed from polyethylene (Herdman, 1949). However, this material does not prevent mechanical obstruction caused by urine settling on the stent tube, which can lead to hydronephrosis. Zimskind et al. (1967) initially delineated *in vivo* silicone ureteral stents (Zimskind et al., 1967). The stent is made up of a straight silicone tube with an open end and a drainage hole on the side. Encrustations of silicone catheters appear to be less prevalent, albeit with higher stent mobility. In order to enhance stent mobility, Gibbons et al. (1974) employed an acorn-shaped silicone sheath at the distal end of the stent, strategically positioned just within the ureteral orifice, thereby facilitating stent fixation within the ureter (Gibbons et al., 1974). Hepperlen et al. (1978) initially documented the utilization of self-retaining single pigtail stents, featuring a proximal pigtail configuration aimed at mitigating distal displacement, but they still have the potential to migrate to the proximal end, making removal difficult (Hepperlen et al., 1978). In the same year, Finney. (1978) reported that double J tubes, with both distal and proximal ends in a “J” shape, greatly reduced the risk of stent displacement, and are still widely used in clinical diagnosis and treatment (Finney, 1978).

**FIGURE 1:**
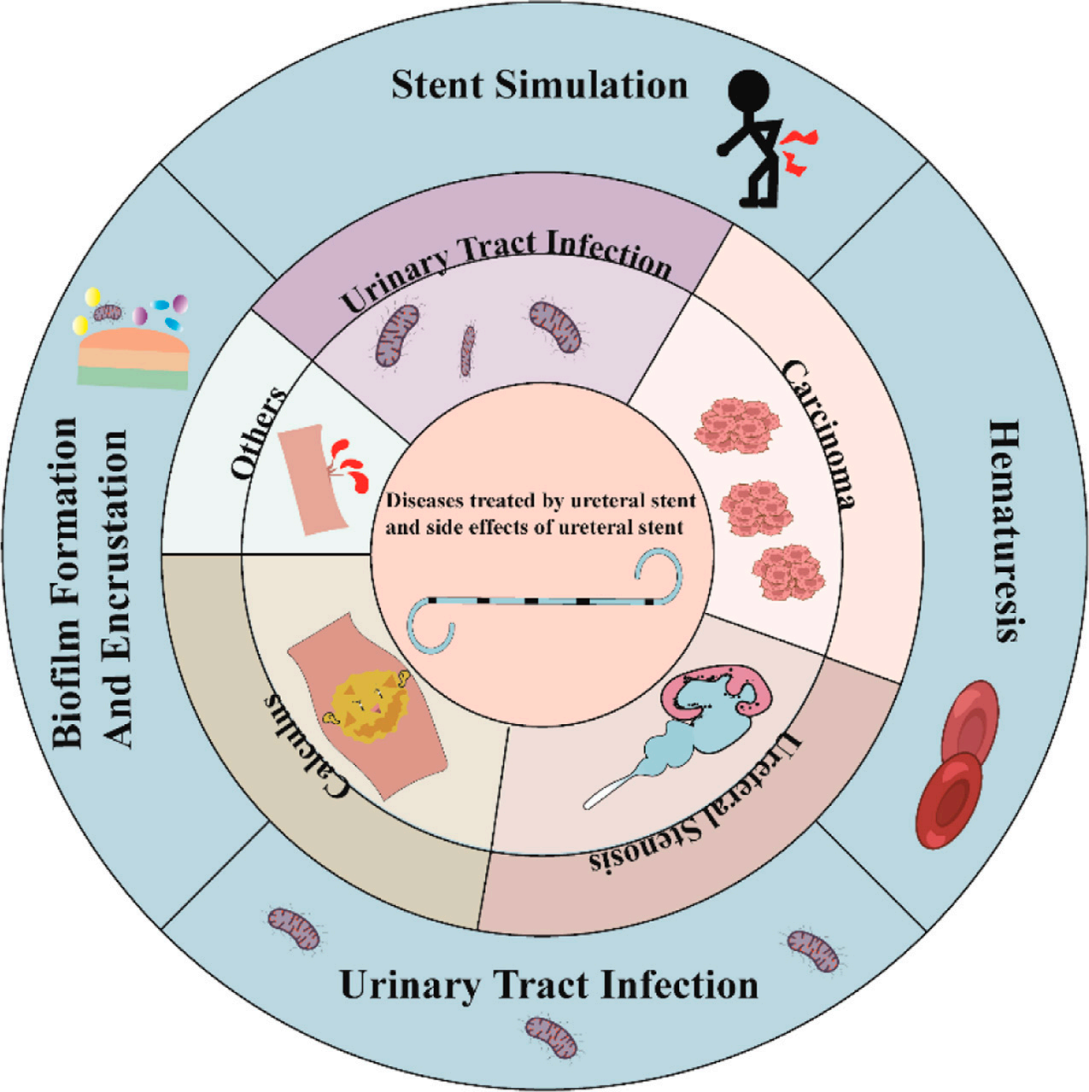
Types of diseases treated clinically with ureteral stents and possible complications after stent implantation.

Currently, diseases such as ureteral tumors, ureteral calculus, and ureteral strictures may lead to renal and ureteral hydronephrosis as well as urinary tract infections, often requiring the use of ureteral stents (as shown in Figure 1). Ureteral tumors represent about 2.65% of genitourinary tumors and have a mortality rate of 3%, according to the 2023 United States Cancer Statistics. The number of ureteral tumor patients in the United States is expected to increase by 500 compared to 2020 data, indicating an upward trend (Siegel et al., 2020; Siegel et al., 2023). The incidence of kidney and ureteral calculus ranges between 4% and 20% (Trinchieri, 1996). An examination of adult Americans unveiled that the incidence of kidney and ureteral stones has shown a continuous rise over the years, escalating from 0.6% in 2005 to 0.9% in 2015 (Tundo et al., 2021). Epidemiological data from the United States and Europe indicate a yearly increase in the incidence of sepsis, with an annual increase of 8.7%. It is noteworthy that 8.6%–30.6% of sepsis cases are related to the urinary tract, and the mortality rate can reach 20%–40% (Martin et al., 2003). Therefore, as the incidence of urological diseases continues to rise, the use of ureteral stents is expected to increase accordingly.

Despite the widespread use of ureteral stents, they are associated with diverse complications, the most common of which are infection, crusting on the stent surface, patient discomfort, and hematuria (Xia et al., 2023). At present, the most commonly used stent tube materials are silicone and polyurethane. These materials require a second invasive procedure with cystoscopy for retrieval and are considered permanent. This secondary surgery increases the risk of postoperative complications such as hematuria and ureteral stricture. For pediatric patients, the removal of the stent requires supplementary general anesthesia. Simultaneously, non-biodegradable ureteral stents are associated with stent retention syndrome (Monga et al., 1995). Over time, the presence of a stent in the body increases the risk of urinary tract infection and the rate of surface crust formation (Klis et al., 2009; El-Hayek et al., 2017). In a recent meta-analysis, the early removal of ureteral stents (i.e., within 3 weeks) was found to significantly mitigate the occurrence of urinary tract infections (Wang et al., 2022). Undoubtedly, the combination of the primary disease and the adverse effects associated with the use of ureteral stents places a significant financial burden on global healthcare systems, presenting substantial challenges to their fiscal resources and long-term sustainability. Researchers are investigating new biomaterials and designs to tackle these issues. Unlike non-degradable ureteral stents, ureteral stents made of biodegradable materials have several advantages, including good biocompatibility that can be broken down into small molecular by-products (Liu et al., 2021), better mechanical properties than currently used polyurethane stents (Antonowicz et al., 2021), the ability of nanoparticles made of biodegradable materials to serve as drug delivery systems (Mehrotra et al., 2023), stent degradability to avoid the risk of second surgery, and the capability to mitigate surface crusting. Biodegradable ureteral stents (BUS) have gained significant attention among scholars due to their potential clinical benefits, making them a promising area of investigation.

The materials used for BUS can be classified into three groups: natural polymers, synthetic polymers, and metallic materials (as shown in Table 1). Regulating the degradation rate and managing the size of degradation fragments are the challenges in the development of biodegradable ureteral stents, which remain in the realm of experimental research. Nevertheless, significant progress has been made in stent design by improving biomaterial properties and biocompatibility. Although still in the experimental research stage, BUS hold promise for future clinical applications. Biodegradable stents have been proposed and shown to be effective in the treatment of benign luminal narrowing in various anatomical structures, including coronary arteries, esophagus, trachea, and others (Saito et al., 2007; Serruys et al., 2010; Zajac et al., 2019).

**TABLE 1:** Comparison of currently used materials for BUS.

BUS materials	Degradation period	Degradation mechanism	Characteristics of BUS	Drawbacks of BUS	References
Alginate	7 days	Benign dissolution	Favorable tissue compatibility	1. Insufficient degradation time	Auge et al. (2002)
				2. Further analysis of human subjects is required	
Alginate	Ureter: 8 days	Even degradation	1. Effective temporary drainage lasting 48 hours	1. 68.2% reported urethral discomfort during the exclusion period	Lingeman et al. (2003a)
	Bladder: 15 days		2. Favorable biocompatibility	2. A 3.4% inadequate degradation rate	
				3. Stent displacement	
Blend of alginate, gelatin, and cold-set jelly	10 days	Even degradation	1. Adjustable *in vitro* degradation rate	1. Rapid *in vivo* degradation rate	Barros et al. (2015a), Barros et al. (2015b), Barros et al. (2016), Barros et al. (2018)
			2. Increased inhibition of bacterial adhesion	2. During *in vivo* degradation, the mechanical performance of BUS decreases	
			3. Uniform degradation		
			4. Effective urine drainage		
			5. High biocompatibility		
			6. Capable of loading ketorolac and anti-tumor drugs		
PLGA (Uriprene™)	First generation:4–10 weeks	Distal degradation is preferred	1. Distal priority degradation prevents debris from blocking the ureter	1. Sudden disintegration of the material	Hadaschik et al. (2008), Chew et al. (2010), Chew et al. (2013)
	Second generation:2–10 weeks		2. Excellent drainage performance		
	Third generation:4 weeks				
Poly(ε-caprolactone) (PCL)/poly(lactic-co-glycolic acid) (PLGA) (LA:GA = 80:20)	5% PCL/PLGA:28 days	Block degradation	1. Controllability of mechanical properties	1. Lack of *in vivo* animal implantation experiments	Wang et al. (2015b)
	15% PCL/PLGA:42 days		2. Flexibility of degradation time	2. Mild inflammatory response observed in rabbit bodies	
	25% PCL/PLGA:56 days				
PCL/PLGA	Begins degradation at 4 weeks, completely degrades within 10 weeks	Gradual degradation from the distal to proximal end	1. The function of gradual degradation from distal to proximal	1. Lack of *in vivo* animal implantation experiments	Wang et al. (2015a)
			2. Effective ureteral drainage	2. Acidic degradation products	
			3. favorable biocompatibility		
Mg-Sr-Ag alloy	The stent continuously degrades during the 12-week implantation period	The surface continuously corrodes and degrades	1. Good cell compatibility and blood compatibility	1. The implantation of the scaffold has a mild impact on the bladder function of experimental animals	Tie et al. (2022)
			2. The adjustability of the degradation cycle of the scaffold	2. The degradation products contain Mg(OH)2, which may lead to an increase in pH	
			3. Higher antimicrobial activity attributed to the release of silver ions		
			4. The scaffold maintains sufficient structural support during the degradation process		
Biodegradable magnesium alloy wires serve as the scaffold for the stent, surrounded by degradable polyurethane material	The stent rapidly degrades after 2 weeks and completely degrades within 5 weeks	Not mentioned	Good biocompatibility	1. Degradation at 4–5 weeks leads to an increase in artificial urine pH	Jin et al. (2021)
				2. Has not demonstrated a perfect antibacterial effect	

**FIGURE 2:**
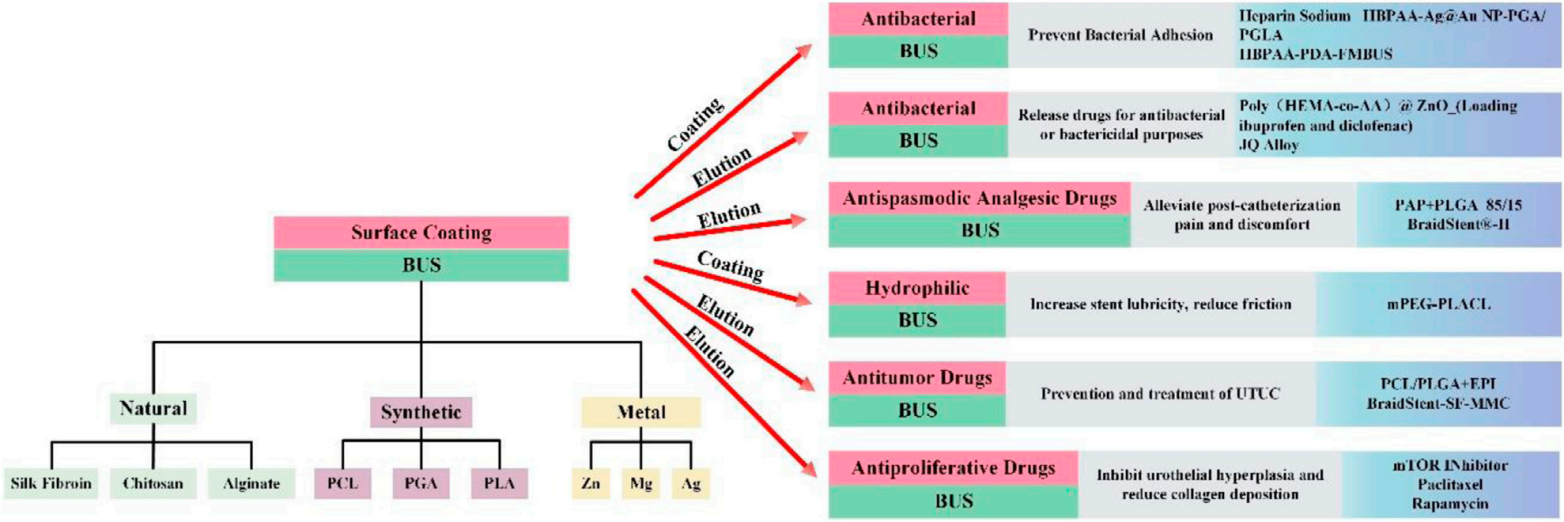
The material classification of BUS and the innovation in clinical functions that can be used in the past 5 years.

Interestingly, in addition to their inherent degradability, BUS can exhibit multiple functionalities when integrated with stent coatings and drug-eluting techniques. This versatility positions BUS to achieve different effects in future clinical applications in the field of urology, offering multiple advantages for the treatment of various diseases. Previous research has explored the incorporation of stent coatings and drug-eluting techniques into non-biodegradable stents, laying the foundation for functional features on BUS surfaces (Chew et al., 2006; Kram et al., 2020). Extensive development and study of drug-eluting, degradable stents have been made in the field of cardiovascular disease (El-Hayek et al., 2017; Kutcher, 2018; Liu et al., 2020). Research on the utilization of BUS in the urinary system is limited, causing delays in the development of ureteral stents compared to the cardiovascular field. This paper summarizes the latest developments in the study of BUS over the past 5 years and explores future clinical applications (as shown in Figure 2). Furthermore, we engage in a comprehensive discussion on the merits and drawbacks of BUS in urology. Additionally, we present our unique recommendations for improving BUS to encourage further research and development in clinical practice.

## 2 Technology applied to the development of BUS

### 2.1 Biodegradable materials for ureteral applications

#### 2.1.1 Natural polymers

Natural biodegradable materials include alginate, gelatin, silk fibroin, and chitosan. In the early 21st century, Auge et al. reported a temporary ureteral drainage stent based on alginate polymers. Although the stent exhibited a short degradation time in porcine experiments (completely degraded in 7 days), it reassuringly degraded in a benign manner, as confirmed by histologic examination, indicating its safety (Auge et al., 2002). Lingeman et al. patented a BUS based on alginate polymers (Lingeman et al., 2003a). *In vivo* experiments confirmed the effective urinary drainage and excellent biocompatibility of the BUS. However, the stent exhibited an inadequate degradation rate of 3.4%, potentially necessitating secondary surgery for retrieval. A decade ago, Barros et al. utilized alginate, cold-setting gelatin, and their blends with gelatin to produce a naturally sourced polysaccharide-based degradable ureteral stent (Barros et al., 2015b). Subsequently, they developed ketoprofen-eluting BUS and anticancer drug-eluting BUS based on this stent (Barros et al., 2015a; Barros et al., 2016). Despite Barros confirming through *in vitro* experiments that the stent could degrade within 14–60 days, with degradation rates controllable by altering the proportion of biodegradable materials, the *in vivo* degradation rate was excessively rapid (10 days), accompanied by a decline in mechanical performance during the degradation process (Barros et al., 2015a; Barros et al., 2016; Barros et al., 2018). Chitosan and silk fibroin have also been explored by other researchers as surface-degradable coatings for ureteral stents, facilitating drug delivery (Ecevit et al., 2022; Soria et al., 2022).

#### 2.1.2 Synthetic polymers

In the development of ureteral stents, the predominant synthetic biodegradable polymers currently include polycaprolactone (PCL), polyglycolic acid (PGA), and polylactic acid (PLA). The first-generation Uriprene stent was composed of 80% LA and 20% GA. It exhibited the advantage of faster distal degradation than proximal degradation, with degradation beginning at 7 weeks and complete degradation at approximately 10 weeks (Hadaschik et al., 2008). The second-generation Uriprene stent began to degrade at 2 weeks and was completely degraded by 10 weeks, while the third-generation stent degraded uniformly within 4 weeks (Chew et al., 2010). However, Uriprene stents were challenged by uncontrolled degradation, leading to sudden material degradation and transient obstruction in animal experiments (Hadaschik et al., 2008; Chew et al., 2010; Chew et al., 2013). Despite significant advances, various PLGA ureteral stents still have drawbacks such as stiffness, high brittleness, poor shape memory, a lack of inherent stability, and multiple fracture events during the degradation process leading to ureteral obstruction (Wang et al., 2015a). Wang et al. utilized dual-nozzle electrospinning technology to design and manufacture a novel stent with a gradient composition of PCL and PLGA (Wang et al., 2015a). The stent consisted of three segments: proximal (25 wt% PCL), intermediate (15 and 25 wt% PCL), and distal (15 wt% PCL). The varying PCL content provided a gradual degradation function from distal to proximal. This stent began to degrade at week 4 and was completely degraded by week 10, with no ureteral obstruction observed in their animal studies. Despite the acidic nature of the degradation products of the PLGA (Lü et al., 2009), experimental results demonstrated no significant difference in pH values between the two groups compared to the control group (Wang et al., 2015a).

#### 2.1.3 Metals

Compared to polymer-based materials for ureteral stents, metallic materials have greater inherent antimicrobial activity and superior mechanical properties (Fu et al., 2020). The enhanced mechanical performance and prolonged lifespan allow patients to reduce the frequency of ureteral stent replacements, maintaining better luminal patency and preventing complications such as recurrent strictures (Abbasi et al., 2013). However, with the prolonged presence of ureteral stents, the inevitable formation of biofilm on the stent wall and biofilm-associated infections are unavoidable (Akay et al., 2007). Hence, biodegradable metal materials have attracted increasing research interest due to their natural degradation advantages and inherent antimicrobial activity (Lock et al., 2014; Zhang et al., 2020). In recent years, magnesium (Mg) and magnesium alloys, zinc (Zn), and zinc alloys have been investigated for the development of degradable ureteral stents (Nguyen et al., 2018; Fu et al., 2020). In 2021, Tie et al. innovatively developed the Mg-Sr-Ag alloy, combining the exceptional mechanical properties of the Mg-Sr alloy with the pronounced antimicrobial activity of silver (Ag). They validated its biocompatibility and impact on the urological system (Tie et al., 2022). Previously, they had preliminarily confirmed the applicability of the Mg-Sr alloy for the manufacture of biodegradable bone fixation devices and demonstrated the pronounced antibacterial activity of magnesium alloys containing silver in an *in vitro* setting (Tie et al., 2013; Tie et al., 2016).

The innovative biodegradable ureteral stent, synthesized from biodegradable polyurethane and magnesium alloy, represents an innovative endeavor. Jin et al. introduced an innovative degradable stent featuring a surface coating of poly-L-lactic acid (PLLA) and poly (lactic-co-glycolic acid) (PLGA) (Jin et al., 2018). Although this stent demonstrated drainage capabilities similar to those of conventional stents, it exhibited renal hydronephrosis when inserted on the degradable side. After 2 years, by capitalizing on the degradation and antimicrobial properties of magnesium alloy in artificial urine, they innovatively designed a biodegradable ureteral stent composed of biodegradable polyurethane and magnesium alloy (Jin et al., 2021). Their research demonstrated negligible cytotoxicity of the degradable stent and excellent biocompatibility. The stent remained nearly intact for the first 2 weeks *in vitro*, followed by rapid degradation and complete degradation within 5 weeks. However, around the 4–5 weeks mark, the stent caused an increase in the pH of the artificial urine, possibly related to the formation of hydroxides from the degradation of the magnesium alloy. The elevated urine pH could promote crystal deposition and lead to incrustation (Tomer et al., 2021). Therefore, further refinement is necessary for the ongoing development of this stent.

### 2.2 Technology for drug delivery systems in BUS

In the past, the mere application of a drug coating to the stent surface could confer functionality to the stent. However, owing to uncontrolled drug release, the outcomes consistently prove unsatisfactory. Amidst ongoing innovation in drug delivery system (DDS) technology, drug-eluting stents have demonstrated the ability to sustain prolonged and effective drug release. The increased stability of the drug-loading function further enhances the suitability of stents for future clinical applications of BUS. Numerous drug delivery systems find current applications in the realm of ureteral stents, encompassing traditional DDS methods (such as hot melt extrusion and polymer soaking in drug solutions), CO_2_ impregnation, nanofibers, and nanoparticles. We have summarized the representative articles corresponding to DDS for BUS, as shown in Table 2. A comprehensive comprehension of the advantages and disadvantages inherent in diverse DDS, coupled with an insight into the contemporary developmental landscape of DDS, is poised to significantly aid the future evolution and clinical transformation of functional BUS.

**TABLE 2:** DDS classification applied to BUS.

Classification of DDS	Advantages	Disadvantages	Application in BUS	参考文献
Traditional DDS (Hot melt extrusion, solvent casting And soak the polymer in a drug solution)	1. The process is simple and easy to make	1. Poor drug control release	1. BraidStent-H® developed by Soria et al., surface-coated heparin for antimicrobial use	Soria et al. (2021b)
	2. Easy for industrial mass production	2. Lack of targeting	2. The BraidStent-SF-MMC developed by Soria et al. is used for the treatment of UTUC	Soria et al. (2023)
		3. Poor water solubility		Edgar and Wang (2017)
		4. Poor sensitivity to drug resistance		
CO_2_ Impregnation	1. Facilitates the loading of drugs into a pre-manufactured implant	1. Adequate solubility in CO_2_ is imperative for the drug	1. Barros et al. pioneered the utilization of this technique in crafting the ketoprofen-eluting BUS	Barros et al. (2015a)
		2. Both the drug loading rate and drug loading efficiency exhibit suboptimal levels	2. They also used the same method to load paclitaxel and doxorubicin into a BUS based on a natural source polymer for the treatment of UTUC	Barros et al. (2016)
		3. The shape of the stent changed after drug loading		Champeau et al. (2015)
Electrospun Nanofibers	1. High encapsulation efficiency for drug loading	1. The active ingredients are unstable	1. Wang et al. used electrospun nanofibers to develop a PCL/PLGA ureteral stent with adjustable drug release and degradation rates of EPI for UTUC	Wang et al. (2019)
	2. Controlled residence time	2. The initial release of the drug		Luo et al. (2012)
	3. The predictability of the delivery rate of encapsulated drugs	3. Residual solvent quantity		Thakkar and Misra (2017)
	4. Better stability	4. Temporary industrial production difficulties		Zamani et al. (2013)
	5. Satisfactory softness and flexibility			
Nanoparticle-Based Drug Delivery Systems	1. The therapeutic efficacy of loaded drugs can be improved by using active targeting of site-specific delivery and protecting normal cells from damage	1. The majority of nanoparticles remain at the stage of cellular and animal testing	1. Gao et al. developed HBPAA-Ag@Au NPs for antimicrobial use	Gao et al. (2020)
	2. Potentially prolonging the duration of action and reducing toxicity	2. Nanoparticles entering the bloodstream may cause adverse effects		Park (2014)
		3. Low drug loading rate		Ding et al. (2019)
		4. Biodegradable polyester nanoparticles are also complex and costly to manufacture		Wang et al. (2023b)
				Wen et al. (2023)

### 2.3 Design for BUS

The current design for ureteral stents involves a bilateral terminal J-shaped tube configuration. While the design of this stent has broad clinical applicability, its use can impede the closure of the ureteral bladder orifice, leading to the occurrence of vesicoureteral reflux (VUR). The stent’s distal end has a curling configuration that, combined with the patient’s movement, may cause irritation to the kidney or bladder, leading to LUTS such as frequent and urgent urination as well as kidney colic. Various stent designs exist to alleviate bladder irritation and prevent VUR, and some individuals have undertaken summarizations of these designs (Domingues et al., 2022). As far as we know, there is no comprehensive summary available for the enhanced design of the ureter specific to BUS.

Presently, the predominant design employed in BUS remains the double J stent, widely favored for its post-implantation stability and resistance to displacement. The synthetic polymer-based gradient degradable scaffold designed by Wang et al. (Wang et al., 2019), the natural polymer scaffold designed by Barros et al. (Barros et al., 2015a; Barros et al., 2015b; Barros et al., 2016; Barros et al., 2018), and the Uriprene stent designed by Chew et al. (2010, Chew et al. (2013), Chen et al. (2022) all adopted D-J design.

To prevent vesicoureteral reflux caused by the double J-tube structure, Lumiaho et al. developed a biodegradable, self-expanding, self-augmenting spiral scaffold with X-ray visibility using poly (L, D-lactide) material (SR-PLA 96) (Lumiaho et al., 2007). The BUS is a 50 mm long stent that forms a double-helix structure. The self-expanding properties of SR-PLA 96 can effectively secure the bracket in place. *In vivo*, the stent is positioned 2 cm above the ureterovesical junction to protect the integrity of the ureterovesical valve mechanism and reduce the risk of VUR. They then demonstrated through animal experiments that the stent’s short ureteral stent design minimizes the use of VUR compared to a double J stent (Lumiaho et al., 2011).

The BraidStent, innovatively devised by Soria et al., boasts a three-part configuration, encompassing a proximal spring coil, a midsection comprising the braidstent, and a distal Nitinol mesh basket for anchoring. The distal end is positioned 2 cm above the ureteral opening to reduce bladder irritation, while the stent prevents ureteral spasms by conforming to the ureteral pathway (Soria et al., 2021b). Nevertheless, the stent deviates from the conventional double J tube design, posing a unique challenge during its implantation for clinicians. Additionally, there is a risk of stent displacement after implantation (Soria et al., 2020).

Wang et al. innovatively engineered a biodegradable mesh ureteral stent tube (Wang Y. C. et al, 2023). The stent length was initially abbreviated by adopting a singular J-shape design. The distal J-shape was eliminated to reduce post-implantation stimulation from the distal coil to the bladder, but this also reduced overall stability. To enhance the stent’s stability, the reticulated ureteral stent was expanded and affixed to the ureter using the balloon dilation technique, thereby increasing its overall stability. The *in vitro* experiments demonstrated the stent’s commendable biocompatibility. Subsequent *in vivo* experiments substantiated the stent’s stability, revealing no instances of displacement post-implantation. However, comprehensive evaluation through animal and clinical trials is still necessary.

Cui et al. (2022) developed a hand-woven PLCL stent and improved the design of the D-J tube to reduce bladder irritation at the distal end of the stent. The distal end of the stent features a singular loop in the J-shaped configuration, which reduces bladder irritation and enhances structural stability.

## 3 Current clinical development of BUS

The main difference between BUS and conventional ureteral stents is their degradability, which can reduce the side effects of ureteral stents after implantation. Domingues et al. summarize four steps for bringing a ureteral stent to market (Domingues et al., 2022). This statement has significant implications for the marketization of BUS. In this section, we shall summarize the stents that have undergone clinical trials or are poised for imminent clinical trials.

Lingeman et al. conducted a phase I clinical trial on the temporary ureteral drainage stent (TUDS) (Lingeman et al., 2003b). Stent drainage was unimpeded within 48 h of placement, and all stents were removed within 1 month from 18 patients with TUDS placement. Although there were no complications related to TUDS with this stent, experimental errors could not be avoided due to the small sample size of this experiment. Larger prospective studies are needed to confirm the clinical feasibility and safety of this stent.

A Phase II clinical trial was then conducted to increase the sample size. They placed TUDS in 87 patients, 68 of whom showed satisfactory results in draining and degrading (Lingeman et al., 2003a). Despite 17 patients exhibiting stent displacement and 3 patients experiencing stent degradation for a duration exceeding 3 months, a notable 89% of the 80 patients conveyed satisfaction with TUDS. The stent can effectively provide temporary drainage for 48 h, making it particularly suitable for patients who require short-term stent retention after ureteroscopy. However, for patients requiring longer retention after major surgery, TUDS may not be suitable. Displacement and partially undissolved stent fragments after stent implantation require reoperation to resolve, and therefore the stent is not used in clinical practice.

The 2nd and 3rd generation of Uriprene stents developed by Chew et al. showed longer degradation time and a certain degree of degradation controllability in pigs that deserve our attention (Chew et al., 2010). The stent is made by the PLGA. The second generation of scaffolds exhibited characteristics of degradation from the distal to the proximal end due to the varying coating thickness. This feature improved the controllability of scaffold degradation. Additionally, the 4-week degradation time of the third-generation scaffolds was consistent with the 4-week time required for clinical disease indications. Therefore, we look forward to future clinical trials of this stent. Unfortunately, despite their good mechanical properties before surgery, the mechanical properties of Uriprene stents during *in vivo* degradation have not been demonstrated.

Zhang et al. reported on a poly (lactic-co-glycolic acid) (PLGA)-woven BUS implanted in dogs. The study demonstrated superior mechanical properties of the PLGA-based BUS compared to non-degradable scaffolds *in vivo* (Zhang et al., 2014). However, there were still reports of stent fragments in the renal pelvis and bladder when using the stents developed by Zhang et al. and Chew et al. despite their degradation (Chew et al., 2010; Zhang et al., 2014). Barros et al. developed a natural BUS using a patented technology that combines the injection process with supercritical fluid technology. The scaffold showed good biocompatibility and degradability, with complete degradation in pigs after 10 days (Barros et al., 2018). Although the stent’s degradation time is short (10 days), which may limit its clinical application, the combination of supercritical technology and BUS is innovative and has potential for drug loading, giving BUS more functions. However, the formulation of the stent may need further improvement before being applied to clinical trials.

The additional antimicrobial capability of the BraidStent®-H developed by Soria et al. has been shown in pigs to reduce bacterial overgrowth as early as possible, although long-term efficacy needs to be improved (Soria et al., 2021a). The stent had a reasonable degradation time (6 weeks). There was no obstructive debris after stent degradation, and VUR was avoided. The stents provide an important reference for the subsequent animal experiments on the functional development of the scaffolds. It is worth looking forward to the testing of the stent in clinical trials.

## 4 Future clinical applications of BUS

### 4.1 Stent encrustation prevention

The incidence of surface stone formation on the ureter is increasing with the prolonged indwelling time of ureteral stents. Encrustation is believed to be a consequence of the development of a urinary conditioning film on the stent’s surface. The formation of the conditioning film begins with the adsorption of urinary proteins onto the stent’s biocompatible material, usually through electrostatic interactions. Increasing the hydrophilicity of the scaffold surface is a strategy to prevent crusting. Enhancing the hydrophilicity of the stent surface can reduce the friction coefficient of the ureteral stent, thereby preventing scab deposition. Ko et al. explored an innovative anti-encrustation coating comprised of monomethoxy polyethylene glycol (mPEG) and 3,4-dihydroxyphenylalanine, applicable to the modification of the surface of ureteral stents to inhibit biofilm formation and scaling in human urine (Ko et al., 2008). However, advancements in this strategy are restricted to the surface of the ureteral stent. Non-biodegradable ureteral stents still encounter the issue of stent-forgotten syndrome. As a result, the research focus has shifted towards developing an optimal biodegradable ureteral stent with an anti-encrustation coating.

Zhang et al. designed a new biodegradable ureteral stent using methoxy poly (ethylene glycol)-block-poly (L-lactide-ran-ε-caprolactone) (mPEG-PLACL) (Zhang et al., 2021). This is due to the flexibility and high elongation at break of polycaprolactone (PCL), combined with the hydrophilic properties of mPEG’s polyether structure, which improve the brittleness and hydrophobicity of poly (L-lactide) (PLA). The combination of mPEG with copolymerized CL and LA products can enhance the hydrophilicity and smoothness of ureteral stents, as well as accelerate their degradation rate. The experimental results suggest that the ureteral stent’s increased surface hydrophilicity accelerates its degradation rate. The water absorption rate of MPEG5-PLACL reaches a maximum of 9.69% after 30 days, followed by a slight decrease. MPEG8-PLACL completely degrades after soaking for 10 days, leading to a decline in mechanical strength due to complete degradation. Furthermore, both *in vitro* and *in vivo* experiments demonstrate that mPEG-PLACL exhibits excellent anti-encrustation effects. The *in vivo* experiment showed a 40% diffuse mucosal hyperplasia rate in the mPEG5-PLACL group, compared to the 100% rate in the PLACL control group, indicating better tissue compatibility. This study confirms that a smooth and hydrophilic surface plays a crucial role in preventing encrustation deposition. However, it is important to note that the *in vivo* encrustation experiment was conducted in the bladder of mice, which differs from the physiological peristaltic function and urine flushing environment present in the ureter. Therefore, further experiments are necessary to confirm the feasibility of this stent in the ureter.

### 4.2 Antibacterial

The bacterial colonization rate of indwelling ureteral stents varies between 42% and 90% (Reid et al., 1992; Kehinde et al., 2004). Both initial contamination during insertion and prolonged indwelling time contribute to the frequent association of stents with urinary tract infections. In a 2021 retrospective analysis, Salari et al. identified positive bacterial cultures from stent specimens as a distinctive risk factor associated with urinary tract infections within 12 months following stent removal (Salari et al., 2021). To reduce bacterial accumulation on stent surfaces, the idea of coating stents and using drug elution has been suggested for biodegradable stents.

**FIGURE 3:**
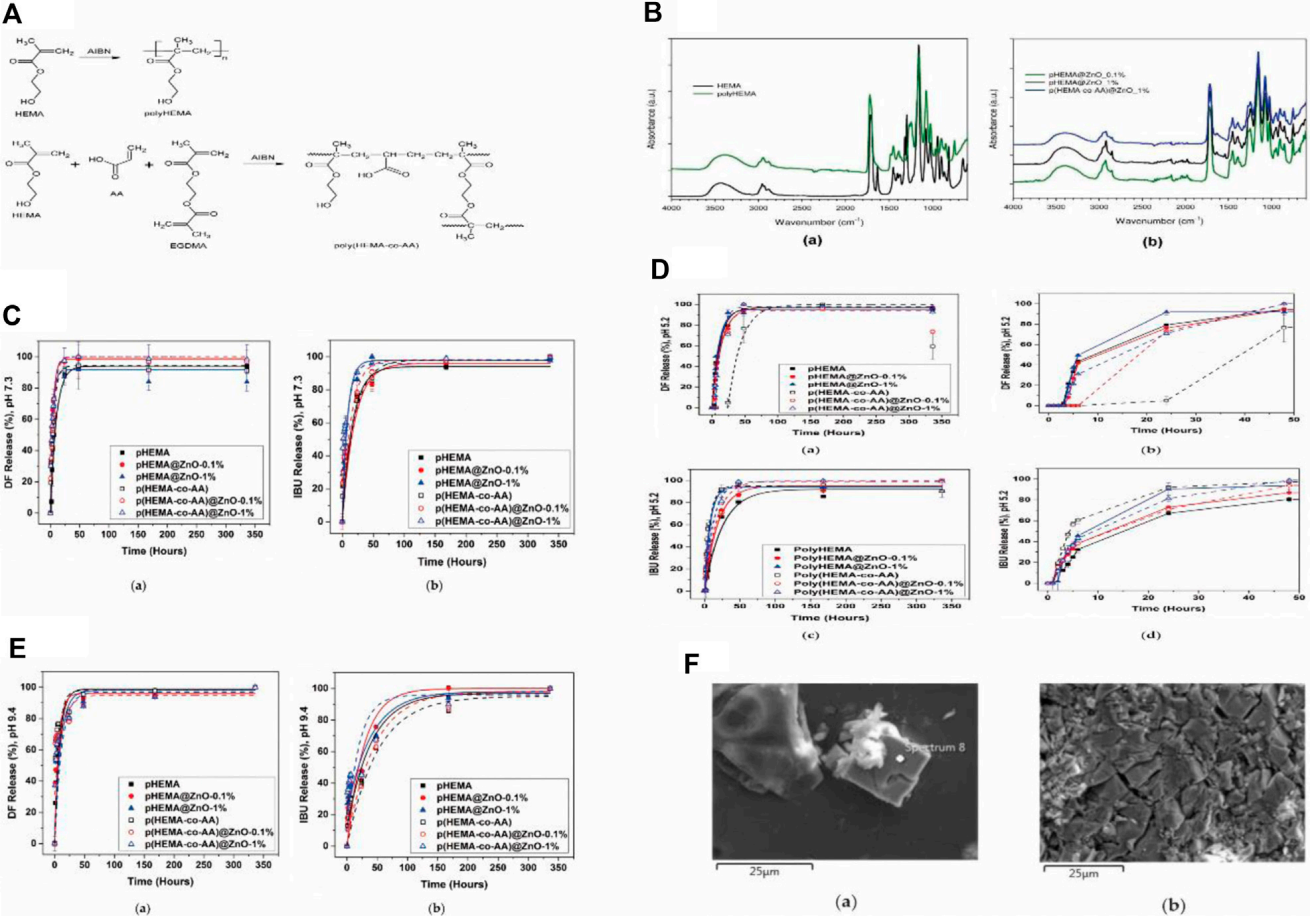
**(A)** The schematic representation of the formation of crosslinked polymers/hydrogel. **(B)** Infrared spectra of HEMA monomer and hydrogels with varying HEMA content, obtained through free radical polymerization, as well as composite samples with different zinc oxide concentrations, were compared. **(C)** Drug release kinetics over time in a simulated physiological urine solution. **(D)** The time-dependent drug release patterns in an acidic simulated urine solution. **(E)** Drug release kinetics over time in an alkaline simulated urine solution. **(F)** FESEM images illustrating the formation of salt deposits and encrustations on the surface of polyHEMA@ZnO after 2 weeks of immersion in artificial urine. Reproduced with permission from ref (Laurenti et al., 2020). CC BY 4.0. Copyright ^©^ 2020 by the authors.

ZnO itself possesses intrinsic antibacterial properties (Garino et al., 2019). However, the release of Zn^2+^ has the potential to trigger cytotoxic effects in healthy cells (Dumontel et al., 2017). Therefore, it is necessary to optimize and monitor their release to ensure biocompatibility. Since ZnO has been shown to readily dissolve in aqueous solutions with a pH below 5.5 or in salt-rich solutions, such as phosphates (Laurenti et al., 2019), employing ZnO-based materials in ureteral stents is not just innovative but also presents a multitude of advantages. It is critical to limit the release of Zn^2+^ from ZnO-based ureteral stents and ensure the drug release challenge. Laurenti et al. prepared hydrogels of poly-hydroxyethyl methacrylate (Poly-HEMA) or crosslinked poly (HEMA-co-AA) copolymers using free radical polymerization, combined with zinc oxide (ZnO) microparticles prepared by a hydrothermal method. The composite carried ibuprofen and diclofenac and was studied in artificial urine (AU) (Laurenti et al., 2020). Their experimental results demonstrated that the combination of ZnO particles with poly-HEMA successfully reduced the release of Zn^2+^, with a decrease of 74.6% and 80.4% in the released Zn^2+^ amount after 3 and 7 days, respectively, compared to pure ZnO particles. This could potentially enhance its cell compatibility. Thermal analysis indicated increased stability of the copolymer as a result of the incorporation of ZnO. Drug release studies conducted In artificial urine under both acidic and alkaline pH conditions, it was demonstrated that the presence of ZnO in the formulation did not adversely affect the hydrogel’s capability to store and release anti-inflammatory drugs. Additionally, ZnO microparticles endowed the composite material with supplementary antibacterial characteristics compared to the pure hydrogel. Furthermore, their release studies indicated that poly (HEMA-co-AA)@ZnO_(0.1%) exhibited good stability and optimal release trends, with modest kinetic constants and restricted burst release. The inherent antibacterial activity of this composite material will be further tested in future studies (Refer to Figure 3 for further details).

Heparin sodium, a naturally occurring glycosaminoglycan, is commonly used as an anticoagulant due to its anti-adhesive properties. In theory, this reduces bacterial adhesion on stents and prevents biofilm formation and encrustation (Al-Aown et al., 2010). Soria et al. found that BraidStent^®^, a type of BUS, was associated with a high bacterial contamination rate, affecting study subjects with a contamination rate as high as 50% (Soria et al., 2020). Consequently, 1 year later, they developed a novel BraidStent®-H, utilizing coating technology to give it a heparin surface coating (Soria et al., 2021b). Their research results demonstrated that BraidStent^®^-H exhibited a programmable and predictable degradation rate, with 91.7% completely degrading within the intended 6 weeks. Although heparin-coated catheters reduced the initial bacteriuria rate, they did not decrease long-term bacteriuria, and the long-term positive asymptomatic bacteriuria rate remained high. Further research is needed to enhance the antibacterial coating to reduce contamination in BraidStent^®^-H. Additionally, the impact of changes in urinary pH values on degradation rates and biocompatibility in patients, considering that experimental animals follow a controlled diet, requires further validation upon human application.

Gao et al. (2020) developed a ureteral stent consisting of a poly (ethylene glycol) (PEG)/poly (lactic-co-glycolic acid) (PLGA) fiber membrane structure with hyperbranched poly (amide-amine) (HBPAA)-terminated silver-coated gold core nanoparticles (Ag@Au NPs). The scaffold’s surface gradient degradation, achieved through continuous peeling, provided the stent with self-cleaning attributes and sustained antibacterial functionality, exposing the HBPAA-terminated Ag@Au NPs inside to eliminate adhered bacteria and proteins. Results from their *in vitro* degradation experiments confirmed the scaffold’s persistent antimicrobial activity, limited release of Ag and Au elements (6.7%, ∼8 μg), coupled with negligible cytotoxicity (L929 cell relative growth rate >80%). In in vivo experiments in pigs, the scaffold exhibited significant absorbable membrane characteristics, reducing inflammation and levels of necrotic cells. No large fragments were noted in the urinary system throughout the degradation process of this scaffold.

Most proteins carry charges and can establish strong adhesive interactions with nonpolar surfaces while demonstrating weak interactions with polar surfaces (Kehinde et al., 2004). By manipulating the material-protein and material-bacteria interface forces, it is possible to enhance repulsive interactions over adhesive attractions. Regarding the material-protein interaction, grafting highly polar polymer coatings with negative or neutral charges onto the scaffold surface can create an anti-protein polar surface. However, due to the lack of antibacterial activity in amphiphilic polymers, they do not prevent biofilm formation caused by bacterial proliferation *in vivo*. At the interface between the material and bacteria, contact-killing surfaces were created by attaching polycationic polymer brushes. However, their antibiofilm properties *in vivo* and durability fell short of expectations. This is due to the fact that host proteins, bacteria, and bacterial fragments are readily drawn by electrostatic forces to engage with positively charged antibacterial surfaces. The resulting biofilm shields the bactericidal moieties, providing a substrate for adjacent thermophilic bacteria, ultimately leading to the loss of surface contact-killing activity. To construct a dual-functional surface simultaneously possessing antibacterial and anti-protein functions, Gao et al. engineered an amino-terminated hyperbranched poly (amidoamine) (HBPAA) with inherent hydrophobicity internally and hydrophilicity externally. This arrangement streamlines the formation of a stronger and denser antibacterial hydrophilic layer (Gao et al., 2021). They further prepared a biomimetic super-hydrophilic patterned (rough) surface through an *in-situ* approach, creating biocompatible polydopamine (PDA) microparticles (MP) on the surface of a biodegradable ureteral stent. They then chemically grafted HBPAA onto the PDA MP, resulting in a highly hydrophilic surface designed for contact-killing. The surface exhibited robust resistance to protein adhesion and achieved synergistic antibacterial and anti-protein adhesion activity. They fabricated the ureteral stent using poly (glycolic acid) (PGA) and poly (lactide-co-glycolide) (GA/LA ratio of 9:1) fibers woven into a structure and converted into a PGA fiber-PGLA membrane structure ureteral stent (FMBUS) through melt processing. Experimental results demonstrated that FMBUS had radial resistance similar to polyurethane ureteral stents (PUUS) used clinically, and no significant decrease in mechanical performance was observed after modification with PDA and HBPAA. Surface charge measurements confirmed that the surface charge of FMBUS could be controlled by adding HBPAA. Results from protein adsorption assessment indicated that increasing amino content could enhance surface hydrophilicity, and the improvement in surface hydrophilicity could provide sufficient repellence to overcome the electrostatic attraction of proteins. *In vitro* and *in vivo* antibacterial experiments proved its effective bacterial inhibition. Cytotoxicity experiments demonstrated that HBPAA-PDA-FMBUS had good biocompatibility. The stent holds great clinical potential for preventing urinary tract infections (UTIs) and stent surface encrustation caused by ureteral stents in the future.

In 2022, Tie et al. fabricated Mg-1.0Sr-0.5Ag (wt.%) alloy (JQ alloy) through semi-solid rheo-extrusion (Tie et al., 2022). Their experimental results demonstrated a 111% increase in ultimate tensile strength for JQ alloy (223.7 MPa) compared to pure magnesium (105.9 MPa). There was a noteworthy improvement in both tensile strength and elongation at the breaking point, ensuring sufficient support performance of the scaffold throughout its entire degradation process. *In vitro* cell compatibility tests indicated that JQ alloy showed comparable cell compatibility and similar blood compatibility to pure magnesium. Pure magnesium has been proven to be a non-toxic implant material (Sun et al., 2019). *In vitro* antibacterial experiments showed that the release of magnesium ions and silver ions from the JQ alloy played a crucial role in its antibacterial effect. The JQ scaffold continuously degraded during the 12-week implantation, reducing the risk of urinary tract obstruction caused by biofilm formation. The reduction in urinary tract obstruction is beneficial for decreasing post-void residual urine volume, which can improve bladder function postoperatively. While this experiment yielded many scientifically significant positive results, additional randomized and prospective multicenter studies, employing diverse animal models, are required to thoroughly showcase the applicability of the alloy. Customization of alloy composition, long-term testing, and comparative studies with different magnesium alloys will be the main directions for future research.

During that very year, Ecevit et al. successfully grafted chitosan (CS) coating polymer chains with three distinct fatty acids (FAs): stearic acid (SA), oleic acid (OA), and linoleic acid (LinA). Afterwards, the CS-FA derivative solution was coated on the surface of a polyurethane (PU) scaffold. This study represents the initial evaluation of these coatings as antibacterial materials on PU-based ureteral stents (Ecevit et al., 2022). The researchers verified the minimal cytotoxicity of the formulated coatings, and the materials exhibited antibacterial potential against various microorganisms. This study provides a new idea for the future use of CS-FA as a surface coating in combination with biodegradable ureteral stents. In order to fully tap the potential of CS-FA as a surface coating for biodegradable ureteral stents, further research and clinical trials are needed.

### 4.3 Carcinoma treatment and prevention

Urothelial carcinoma can occur in both the upper and lower urinary tracts. The upper tract includes the ureters and the renal pelvis-calyceal system, while the lower tract comprises the urethra and bladder. It is a multifocal disease with a tendency for local recurrence and metastasis. According to the 2023 US cancer statistics, bladder urothelial carcinoma is the fourth most common cancer in males in terms of incidence and the eighth most common in terms of mortality. Bladder tumors make up 90%–95% of urothelial carcinomas, which are the most common tumors in the urinary tract. In contrast, upper tract urothelial carcinoma (UTUC) is less common, with ureteral tumors accounting for only about 2.65% of urinary system tumors and a mortality rate of 3% (Siegel et al., 2023). UTUC is stratified into low-grade and high-grade risk levels. Low-grade UTUC patients benefit from nephron-sparing surgeries. After the surgery, it is crucial to place a ureteral stent to support the ureter and ensure the smooth flow of urine into the bladder. The most critical postoperative challenge is preventing tumor recurrence and managing the side effects associated with ureteral stent placement. To prevent the recurrence and progression of low-grade UTUC, drug instillation therapies can be used. Adjuvant instillation of mitomycin C (MMC) has shown promise in reducing the likelihood of urothelial recurrence and progression in individuals diagnosed with low-grade UTUC, offering hope for survival without nephroureterectomy (Metcalfe et al., 2017). However, administering intraluminal chemotherapy infusion is challenging due to the washout effect caused by urine produced by the kidneys and the limited storage capacity of the upper urinary tract, in comparison to bladder instillation (Jung et al., 2019). Patients with low-grade UTUC who undergo nephron-sparing surgery may not benefit from adjuvant chemotherapy instillation procedures, resulting in poorer outcomes. Biodegradable ureteral stents loaded with anti-tumor drugs offer a clinical prospect for tumor treatment and prevention by maintaining long-term effective drug concentrations at the lesion site. Furthermore, their self-degradation capability can overcome the drawbacks of traditional ureteral stents.

**FIGURE 4:**
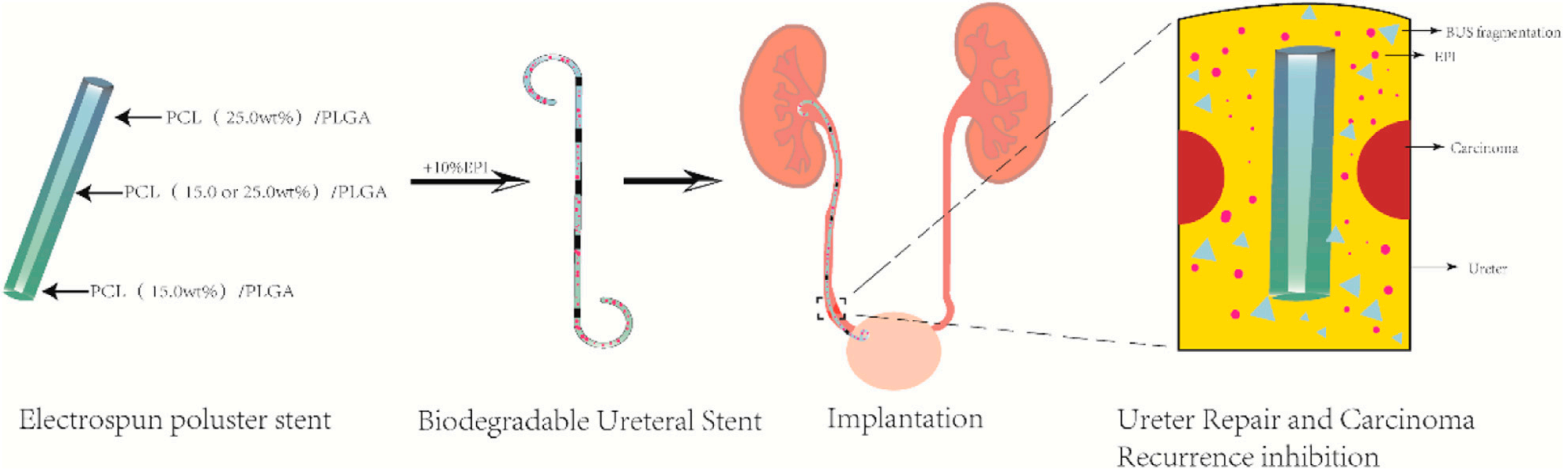
Illustration depicting the *in vivo* application of a ureteral stent to prevent the recurrence of upper urinary tract tumors and facilitate ureter repair.

Wang et al. developed a biodegradable ureteral stent using electrospinning technology for the targeted delivery of anticancer drugs (Wang et al., 2019). Varying concentrations of the anticancer drug epirubicin (EPI) were loaded onto a gradient-degradable PCL/PLGA scaffold with 15.0% and 25.0% PCL, which was designed for treating UTUC (as shown in Figure 4). The various scaffolds displayed different degradation rates and kinetics of EPI release. The experimental results showed that the PCL/PLGA scaffold had the characteristics of sustained and controllable degradation. The degradation rate of the scaffold could be adjusted by modifying the PCL ratio, and reducing the PCL content could accelerate both drug release and degradation rates. The study demonstrated the effectiveness of the scaffold in inhibiting the growth of bladder tumor cells both *in vitro* and *in vivo*. Additionally, the *in vivo* application of all EPI-loaded scaffolds showed no apparent systemic toxicity. The research suggests that using electrospun polyester scaffolds with gradient degradation is a promising approach to support and repair ureteral drainage while preventing the proliferation of residual tumor cells.

**FIGURE 5:**
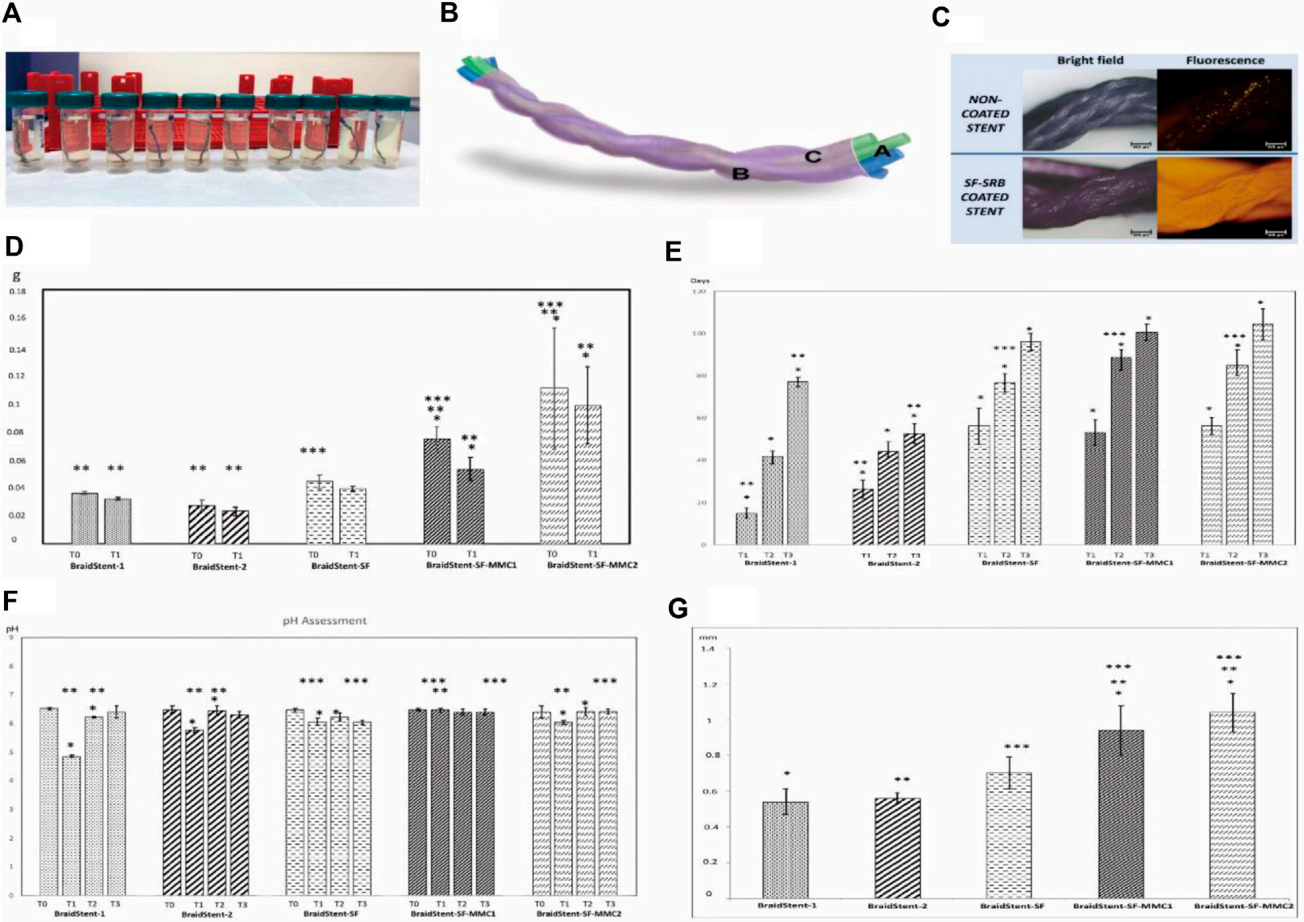
**(A)** Fragments of ureteral stents submerged in artificial urine on the 6th day. **(B)** Illustration of BraidStent-SF-MMC. **(C)** Evaluation of SF coating using SRB fluorescent dye. **(D)** Evaluation of the reduction in weight (in grams) from the initiation of the study (T0) to the commencement of stent degradation (T1). **(E)** Degradation rate assessment (days) **(F)** Assessment of pH throughout the study until complete stent degradation. **(G)** Initial (T0) stent thickness in the experimental groups (in millimeters). Reproduced with permission from ref (Soria et al., 2022). CC BY 4.0. Copyright ^©^ 2022 by the authors.

Soria et al. developed a coating for the biodegradable ureteral stent BraidStent^®^ using silk fibroin protein. They then loaded two formulations of mitomycin C into the polymer matrix to evaluate the stent’s degradation rate, released mitomycin C concentration, and changes in pH and weight (Soria et al., 2022). The BraidStent^®^ was divided into two groups: BraidStent-1, a long-term woven stent containing GlycomerTM 631 (a Biosyn suture produced by Covidien in Minneapolis, Minnesota, USA) and poly (4-hydroxybutyrate-co-hydroxyvalerate) (PGA); and BraidStent-2, a short-term woven stent containing GlycomerTM 631 and PGA. The polymer composition ratio for each stent remained constant during its manufacturing process: GlycomerTM 631 (54%); PGA and poly (4-hydroxybutyrate-co-hydroxyvalerate) (46%). After degradation in AU, BraidStent-2 was chosen because it complies with degradation standards within the first 7–8 weeks and has weaker medium acidification caused by degradation products compared to BraidStent-1. Silk fibroin coating and MMC formulation addition were performed on BraidStent-2. The results confirmed that the silk fibroin protein matrix can coat the biodegradable stent and release mitomycin C for 6–12 h in artificial urine. Furthermore, the BraidStent-SF-MMC exhibited a significantly delayed degradation rate compared to the uncoated biodegradable stent. The degradation time was extended from 6–7 weeks to 13–14 weeks. Although the degradation process caused a significant decrease in pH, it remained within the normal range for humans. The authors proposed that silk fibroin enables processability in multiple forms, including gel, membrane, coating, and stent. In 2023, Soria et al. conducted further evaluation of BraidStent-SF-MMC in a study involving 14 female pigs with single kidneys (Soria et al., 2023). The experimental results indicate that the stent was able to release mitomycin within the first 12 h. However, during the first and third weeks, 28.5% and 7.1% of the animals, respectively, experienced the release of obstructive ureteral coating fragments, which may have been caused by a pH < 7.0. Furthermore, a concerning 21% ureteral stricture rate was observed between weeks 4–6. In contrast to the previously mentioned *in vitro* degradation time, the stent completely degraded *in vivo* within 6–7 weeks (Refer to Figure 5 for further details).

### 4.4 Postoperative discomfort and pain management

Joshi et al. developed and validated the Ureteral Stent Symptom Questionnaire (USSQ). The questionnaire revealed that over 80% of patients with benign conditions who undergo stent implantation experience symptoms of irritative voiding, pain, and discomfort (Joshi et al., 2003). Harper et al. discovered a noteworthy rise in stent-related symptoms among patients on the first day after surgery. However, there was an approximately 50% reduction in pain intensity from postoperative day 1 to day 5, yet the interference caused by pain continued to rise (Harper et al., 2023). Pharmacological intervention is often required to manage lower urinary tract symptoms (LUTS) and stent-induced pain. Treatment options include alpha-1 receptor blockers and antimuscarinic agents such as solifenacin or a combination of solifenacin and tamsulosin (He et al., 2016). To alleviate the pain caused by ureteral smooth muscle contractions resulting from stent implantation, spasmolytic drugs such as papaverine hydrochloride (PAP) are used. This helps to reduce patient discomfort.

**FIGURE 6:**
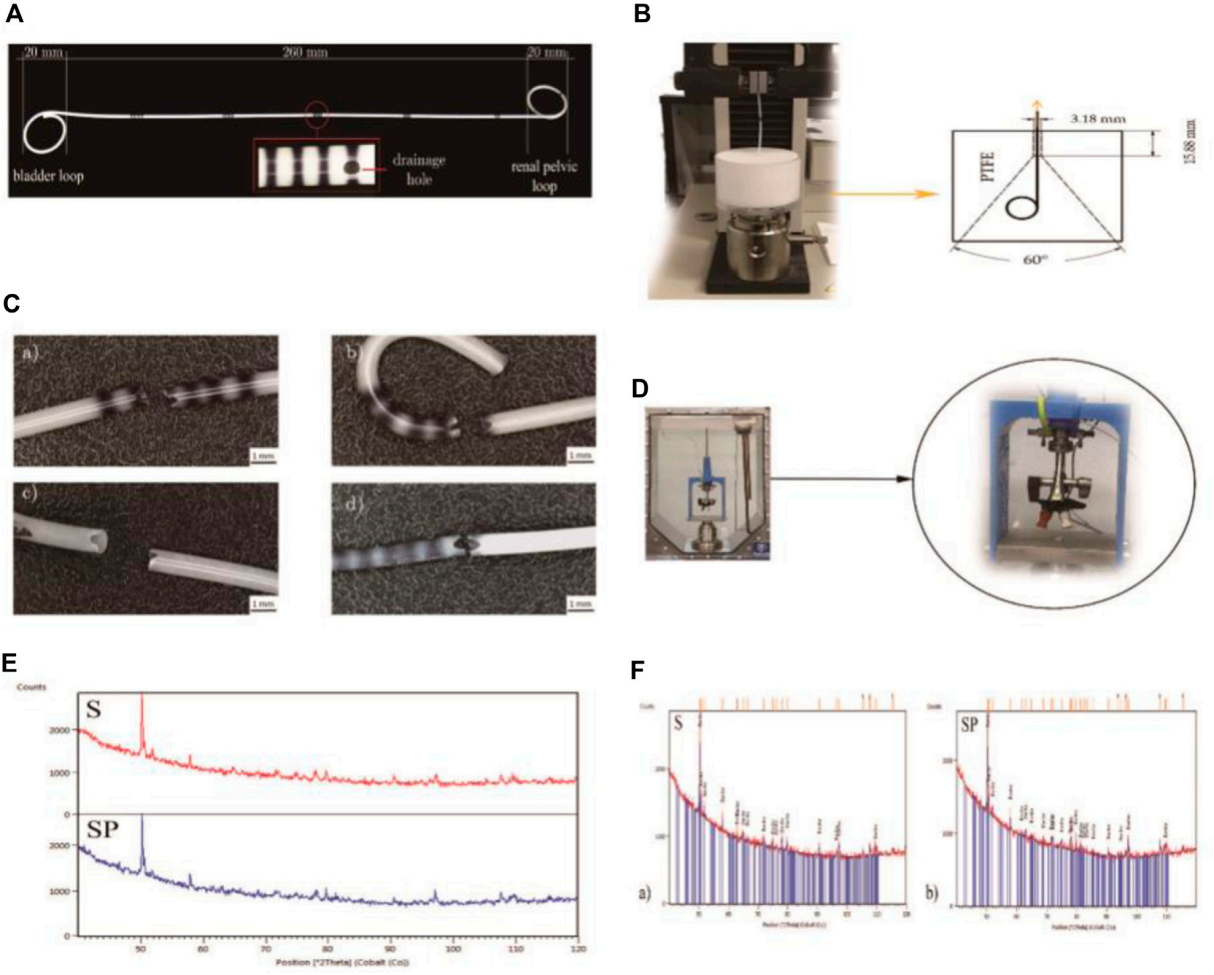
**(A)** Polyurethane Double-J ureteral stents. **(B)** Apparatus for evaluating the retention strength of the distal and proximal ends of ureteral stents. **(C)** Ureteral stents post static tensile testing. **(D)** Experimental setup for conducting dynamic frictional force tests. **(E)** X-ray diffraction (XRD) patterns for the polyurethane stent (S) and the coated stent (SP). **(F)** XRD patterns of the barium sulphate (BaSO4) standard for the ureteral stents. Reproduced with permission from ref (Antonowicz et al., 2021). CC BY 4.0. Copyright ^©^ 2021 by the authors.

Antonowicz et al. (2021) conducted a study on polyurethane ureteral stents coated with poly (lactic-co-glycolic acid) (PLGA) 85/15 containing papaverine hydrochloride (PAP). The research shows that stents coated with PLGA+PAP have better strength properties and a lower dynamic frictional force compared to polyurethane stents, without compromising fluoroscopic visibility (Refer to Figure 6 for further details).

Soon afterwards, they investigated the impact of artificial urine on the physical and chemical properties of PLGA+PAP-coated stent tubes, building upon the foundation of previous studies on the stent tubes (Antonowicz et al., 2022). The study showed that applying PAP coating increased surface hydrophilicity, which could make stent implantation easier. The amount of PAP in the coating decreased to 77% on the 10th day and further to 36% after 20 days. The early release of PAP was advantageous as it caused ureteral dilation, reducing patient discomfort. However, stents coated with PLGA+PAP exhibited increased surface roughness, which made them susceptible to the deposition of artificial urine components. Therefore, further refinement is necessary for the developed stents, with a focus on achieving uniform drug distribution, appropriate polymer degradation time, and a smooth, even drug-loaded layer. Additionally, *in vivo* studies are necessary to confirm the effectiveness of the incorporated drug, as the experiments were limited to *in vitro* validation.

**FIGURE 7:**
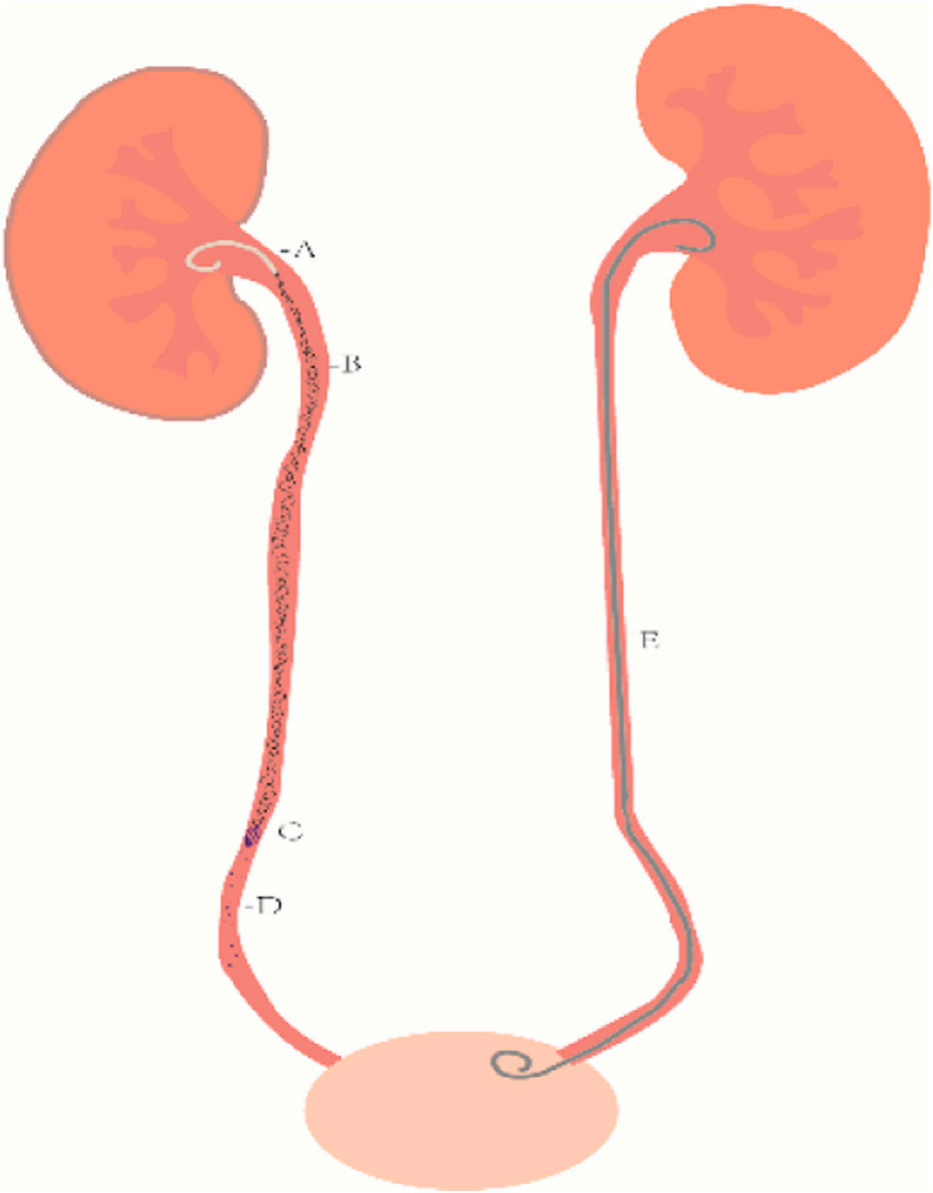
Illustration of the upper urinary tract BraidStent^®^-H. **(A)** proximal pig tail. **(B)** Central portion with a four-line braid. **(C)** Distal anchoring system with a rounded four-line braid. **(D)** Degradation fragments. **(E)** Clinically common non-degradable ureteral stent.

In the same year, Soria et al. introduced the BraidStent®-H. Unlike traditional ureteral stents, the distal end of BraidStent®-H is positioned 2 cm above the ureteral orifice, avoiding bladder irritation and the consequent lower urinary tract symptoms (LUTS). The schematic representation of the stent design is illustrated in Figure 7 (Soria et al., 2021b). Existing literature supports the idea that the highest nerve density in the human ureter is located in the distal region (Vernez et al., 2017). Therefore, BraidStent^®^-H, by expanding along the ureter, can reduce ureteral spasms, consequently minimizing the onset of acute pain. In their study, during the 6-week implantation period, the BraidStent^®^-H significantly mitigated the side effects associated with conventional stents, as it did not exhibit VUR or induce bladder irritation.

### 4.5 Prevention and treatment of ureteral stricture

Ureteral strictures are frequently caused by inflammation, stones, trauma, surgical scars, and iatrogenic injuries. Iatrogenic factors account for approximately 75% of cases (Delvecchio et al., 2003). Urothelial cells, like vascular endothelial cells, respond to injuries by inducing excessive proliferation of fibroblasts and smooth muscle cells. This leads to progressive scarring and eventual ureteral stricture. Severe strictures can cause obstruction by compressing and deforming ureteral stents. Therefore, the idea of drug-eluting stents with anti-fibrotic properties has been suggested.

The use of biocompatible stents loaded with anti-proliferative drugs, such as paclitaxel, rapamycin, or sunitinib, allows for sustained and controlled drug release to prevent cell proliferation. Research has extensively focused on drug-eluting stents for cardiovascular applications, with studies indicating improved vascular healing with such coatings (Steigerwald et al., 2012). In comparison to cardiovascular stents, drug-loaded biodegradable ureteral stents offer the advantage of partial excretion of drug and polymer fragments through urine after drug release and stent degradation. In 2011, Will et al. (2011) assessed the influence of paclitaxel on the proliferation of ureteral smooth muscle cells and collagen production. The study showed that paclitaxel effectively inhibits smooth muscle cell proliferation and reduces collagen production. Additionally, it was found to be non-toxic to smooth muscle cells.

Kram et al. (2020) investigated the effectiveness of paclitaxel-coated stents in inhibiting tissue proliferation in a rat model. The study involved performing ureteral anastomosis in rats and inserting either coated or uncoated stents. After 28 days, the rats were euthanized, and the ureters were examined histologically and immunohistochemically. The results showed a significant reduction in the proliferation of urothelial cells in animals with paclitaxel-coated stents. However, there were no significant differences in stent patency, migration, or biofilm formation between coated and uncoated stents. The drug released from the stent was limited to the ureter, maintaining its concentration within a non-toxic range. Therefore, their experiments support the idea that coated stents can effectively reduce tissue proliferation in ureteral anastomosis and prevent postoperative stricture formation.

Afterwards, Ho et al. investigated the impact of a drug-eluting biodegradable stent (DE stent) loaded with an mTOR inhibitor (mTORi) on ureteral stricture associated with thermal injury in rabbits (Ho et al., 2021). The DE stent is a recognized device for inhibiting coronary artery restenosis. Their study demonstrated that treatment with a biodegradable stent releasing rapamycin significantly alleviated thermal injury-induced urinary obstruction and reduced the levels of fibrotic proteins.

It is not a mere coincidence that Hu et al. developed a dual-layer drug-eluting ureteral stent with rapamycin loaded onto an optimized 75/25 poly (lactic-co-glycolic acid) (PLGA) and polycaprolactone (PCL) dual-layer polyurethane ureteral stent (Hu et al., 2023). This dual-layer stent prevents the sudden loss of drugs and maintains a sustained delivery time. Their results indicate a significant effect of the drug-eluting stent in inhibiting ureteral scar proliferation compared to the bare stent. Moreover, the dual-layer stent with PCL as the outer layer exhibits a slower release rate of rapamycin than a single-layer PLGA stent. The drug release rate of PLGA/drug/PCL gradually increases over 21 days, providing a consistent inhibitory effect on ureteral stenosis.

The anti-proliferative drug-coated stents, upon future commercialization, are poised to not only prevent postoperative strictures caused by scar formation after reconstructive surgery but also to preclude urothelial proliferation reactions in patients with non-anastomotic ureteral openings. This development not only addresses the risk of postoperative complications in reconstructive surgery but also mitigates urothelial proliferation concerns in patients with yet-to-be-anastomosed ureteral openings.

## 5 Limitations/shortcomings and strategies for improvement

After years of dedicated research, significant progress has been made in the field of biodegradable ureteral stents (BUS). Many BUS have demonstrated improved performance through continuous efforts for refinement. However, BUS currently still has three major limitations. In the fabrication of scaffolds, it is necessary for researchers to select materials with high biosafety to make BUS. Meanwhile, it is worth exploring how to use processing technology suitable for standardized mass production to produce scaffolds with excellent performance. BUS degradation should be controlled, and degradation debris should not be too large to avoid urinary obstruction. The balance between the ideal degradation time for scaffolds and adequate mechanical properties when degraded remains a challenge. Drug release safety cannot be ignored when developing stents. *In vitro* experiments, the standardization of *in vitro* degradation results may be hampered by the lack of a uniform standard for simulating *in vitro* hydrodynamic conditions and simulating artificial urine to test BUS. Thus, it becomes imperative to develop appropriate standard detection methods to obtain reliable and comparable results, thus contributing to new and better advances in this area. In terms of *in vivo* experiments, most of the current *in vivo* experiments are mainly based on animal experiments. Clinical experiments are different from animal experiments, and human subjects cannot achieve the same control variables as experimental animals. Human subjects have differences in diet, physique, etc. Therefore, the safety, degradability and functional efficacy of BUS scaffolds still need to be confirmed by clinical trials with a large sample size. Here, we present innovative solutions to address the challenges posed by the development of BUS, which may inspire future BUS development.

### 5.1 Material innovation

Currently, the predominant scaffolds consist primarily of materials such as PLGA and PLCL. These polymers are mainly bulk degradation modes. An ideal characteristic of this mode is that, under optimal conditions, the mass of the sample remains constant during the initial stages of degradation while the molecular weight continues to decrease. The structure of the sample collapsed, resulting in small fragments. Conversely, the alternative form of degradation is surface corrosion, which initiates from the material’s surface and is characterized as “layer-by-layer degradation.” The identification of materials possessing surface corrosion properties for constructing BUS could address the issue of sudden support cracking. At the same time, materials whose degradation mode is dominated by surface dissolution during the degradation process are more suitable to be used as drug carriers in the constant rate release drug delivery system (Zhang et al., 2008).

Poly (trimethylene carbonate) (PTMC) has recently gained widespread attention. TMC exhibits unique degradation behaviors, including the production of non-acidic degradation products, resistance to non-enzymatic hydrolysis, and a surface erosion mechanism induced by enzymatic degradation (Fukushima, 2016). Yang et al. synthesized a biodegradable network based on PTMC through the ring-opening polymerization of TMC and CL. The network’s glass transition temperature was found to be below physiological temperature (37°C), suggesting its rubbery nature for *in vivo* applications (Yang et al., 2013). It is anticipated that in future development, there will be intensive studies on ureteral stents that are based on the unique degradation properties of PTMC and *in vivo* rubber properties.

The degradation rate of BUS is primarily governed by the choice of the stent’s material, allowing for future customization of degradation times based on the selected combination of biodegradable biomaterials and their inherent properties. Yang et al.'s *in vitro* degradation experiments revealed that crosslinking significantly influences degradation behavior. The crosslinked PTMC network is less sensitive and more resistant to lipase degradation, but it exhibits superior shape stability compared to the non-crosslinked PTMC network. Additionally, the degradation of the crosslinked PTMC network is faster in enzymatic degradation than hydrolytic degradation. In in vivo degradation experiments, crosslinking can be adjusted by modulating crosslink density and incorporating CL content to regulate the PTMC degradation rate (Yang et al., 2016). Urine contains amylase and uricase (Stump et al., 1986; Zheng et al., 1991). Therefore, the enzymatic properties of PTMC may have future applications in urology.

Liu et al. prepared a biodegradable elastic PTMC/PLC (80/20) networked ureteral stent (Liu et al., 2021). The *in vitro* degradation experiment of the scaffold demonstrated that the scaffold underwent relatively rapid erosion, facilitated by porcine pancreatic lipase. Surface erosion was observable under SEM, and the scaffold retained adequate tensile strength throughout the degradation process. At the same time, the network is biocompatible. This experiment was lacking in in vivo degradation experiments and clinical trials of this scaffold.

PTMC may become one of the materials for future development in biodegradable ureteral stent (BUS) research, and we are currently conducting research on materials for ureteral stent tubes. Perhaps in the future, copolymers with distinct properties in enzymatic and hydrolytic degradation can be utilized to craft stents with ideal degradation times.

Scaffold surface crusting is a significant issue in clinical stent implantation due to the formation of biofilms composed of bacteria that can cause catheter and scaffold scaling. Current research is primarily focused on slow-release antibiotic coatings, but resistance remains a concern. Biocompatible antifouling coatings, such as novel biomaterials with properties like antimicrobial peptide coatings and brush materials, may be crucial for future BUS development. Yao et al. demonstrated that scaffolds containing antimicrobial peptides exhibit good biocompatibility and inhibit bacterial growth and biofilm formation *in situ*. This ultimately reduces the deposition of struvite and hydroxyapatite crystals, both *in vitro* and *in vivo* (Yao et al., 2022).

Polyvinylpyrrolidone iodine (PVP-I) is a hydrophilic polymer material that can help prevent urinary tract infections. This antibacterial material is not an antibiotic and may significantly reduce the production of drug-resistant bacteria. Khandwekar et al. found that the deposition of struvite and hydroxyapatite, the main components of urinary tract calculus, was significantly reduced on PVP-I modified polyurethanes, especially the reduction of hydroxyapatite calculus (Khandwekar and Doble, 2011). The support exhibits excellent anti-adhesion and anti-scaling properties, as well as remarkable durability. However, the stent has not been extensively studied *in vivo*. Therefore, it is imperative to investigate its effects on animal models and its application to BUS.

The clinical functionality of the stent is primarily manifested by the drug delivery system incorporated into the stent. Nanoparticles made from biodegradable polyester have gained significant attention as a drug delivery system (Cheng and Pun, 2015). Nanoparticles have the advantages of actively targeting and protecting normal cells from damage and improving the therapeutic effect of loaded drugs (Park, 2014).

Owing to the existence of blood vessel barriers, the penetration of nanomaterials into tissues occurs at a slower rate compared to small molecule drugs (Poste et al., 1982). In order to reduce the clearance of the reticuloendothelial system (RES), the development of the cloaking function of the nanocarriers has become a research hotspot. Wen et al. (2023) reviewed the cloaking nanocarriers in detail and proposed the concept of the “pseudo-stealth effect.” In future developments, the antifouling or invisibility properties and biocompatibility of the polymer coating of the scaffolds should be key to future BUS functional development and translational clinical applications.

### 5.2 Manufacturing process innovation

The innovation in the BUS manufacturing process has the potential to enhance both the physical and chemical properties of the scaffold. 3D-printed stents have the capability to design geometric shapes that are specific to individual patients and possess complexity, thereby achieving optimal mechanical properties. Moreover, it is feasible to 3D-print smart materials endowed with shape memory.

At present, 3D printing has been successfully applied to the manufacture of trachea stents and cardiovascular stents (Zopf et al., 2013; Sousa et al., 2022). Although this technology has been used in the development of non-degradable ureteral stents (Lee et al., 2022), However, there are still few researches on the application of 3D printing technology to BUS production.

Chang et al. (2020) applied 3D printing technology to the production of BUS, and they confirmed that the scaffolds produced by 3D printing technology had suitable mechanical properties and degradation rate.

Through the advancement of 3D printing technology, the biodegradable BUS survival approach emerges as a means to personalize and facilitate the mass production of ureteral stents. The successful integration of this technology with BUS is anticipated to contribute significantly to alleviating patient discomfort.

### 5.3 Tracking and follow-up capability innovation

In clinical practice, X-rays and computed tomography (CT) are considered the most common and least invasive tools for observing the condition of medical implants in the body (Samuel et al., 2015). Given the challenges of stent breakage and displacement in BUS, the tracking and follow-up capabilities of the stent hold paramount significance. Consequently, the development of BUS with suitable radio-impermeability warrants our attention.

Chang et al. introduced three radiopaque agents into polyglyceryl sebacate acrylate (PGSA), including barium sulfate, bismuth basic carbonate (BiO)_2_CO_3_), and bismuth oxychloride (BiOCl) (Chang et al., 2020). Experimental findings indicate that the mechanical properties of bismuth oxychloride-embedded PGSA (PGSA-BIOCL) at an embedding concentration of 25 wt% surpass those of PGSA-Baso_4_ and PGSA-(BiO)_2_CO_3_. Furthermore, it demonstrates high linearity in non-transmittance. However, a linear correlation between the transmission linearity and the embedded content cannot be established. *In vitro* experiments reveal that PGSA-BiOCl25wt% exhibits commendable biocompatibility and degradability.

The advancement of materials and technologies amenable to tracking through rapid and non-invasive imaging techniques is poised to play a pivotal role in the initial development and clinical application of BUS. Moreover, the follow-up of various diseases is expected to be facilitated.

## 6 Future prospects

Since non-degradable stents are still widely used in clinical practice, the complication rate of stents is an issue that clinicians need to weigh, so BUS research is urgently needed for clinical application. The ideal BUS in the future should have: (1) good biocompatibility; (2) moderate mechanical properties to ensure ureteral drainage comfort; (3) complete biodegradation with no obstruction or biological damage caused by degradation products; (4) good ability to prevent migration; (5) diversity of stent functions; (6) good tracking ability. The “ideal” BUS has not yet been produced. The production of stents requires in-depth communication between various disciplines, including but not limited to medicine, materials science, pharmacy, molecular biology, etc.

The controlled degradation time of BUS can be adapted to physicians’ different clinical decisions. For example, in cases of malignant tumors, clinicians currently require long-term placement of ureteral stents with periodic replacement, which is inconvenient for both healthcare professionals and patients. In 1 day, BUSs with extended degradation times may address this clinical treatment challenge. Conversely, short-degradation BUS may be more suitable for post-operative patients requiring short-term stent placement.

In addition to the versatile clinical applications for treating diseases with drugs, the surface coatings and drug-eluting capabilities of BUS can also facilitate localized drug delivery. In comparison to systemic administration, local drug delivery offers several advantages, such as reducing drug dosage and minimizing side effects. This may contribute to enhancing treatment efficiency and reducing the harm caused to patients by medications.

The fragments resulting from BUS degradation need to be small enough for easy expulsion from the ureter, avoiding the risk of ureteral obstruction. Simultaneously, the degradation products of BUS should not induce changes in the urinary environment within the human body. In the BUS developed by Jin et al. (2021), an increase in the artificial urine’s pH was observed as a consequence of degradation. Hou et al. (2017) demonstrated that cross-linked poly (trimethylene carbonate) networks (PTMC-Ns) offer adjustable degradation rates, enhanced shape stability, and the advantage of producing non-acidic degradation by-products. Perhaps, in the future, the favorable biocompatibility and non-acidic degradation products of PTMC will be utilized in the development of BUS.

In the future, BUS will reduce stent infection, stent crusting, hematuria, pain and other complications caused by stent implantation. It will also reduce the time of stent implantation, reduce the frequency of stent replacement, and prevent stent forgetting. Further exchanges between medicine and other disciplines can promote the progress of human society.

## 7 Conclusion

This review summarizes the functions, clinical transformation, shortcomings and future prospects of BUS, and puts forward innovative suggestions to solve the related deficiencies. Currently, BUS scaffolds are unable to attain the ideal characteristics primarily due to challenges in controlling degradation time, mechanical properties, and degradation fragments. Additionally, researchers encounter challenges in achieving the requisite drug release performance for the subsequent follow-up and functionalization of scaffolds. In order to solve the above problems, we believe that innovations need to be made in BUS manufacturing materials, BUS manufacturing processes, selection of BUS coating and development of tracking and follow-up functions, including PTMC as a scaffold material, the application of 3D printing technology, nanoparticle drug delivery and the use of developer. Additionally, the development of functionalities related to stent coatings and drug elution remains a current research focus. At the same time, the interdisciplinary cooperation and learning of biomaterials and medicine are inevitable in the development of BUS in the future, and the in-depth exchanges and communication between clinicians and material science experts will accelerate the development of BUS. In conclusion, BUS holds promising applications in the field of urological surgery.
